# Editorial: The dark or bright side of entrepreneurship

**DOI:** 10.3389/fpsyg.2022.1041699

**Published:** 2022-10-11

**Authors:** Yenchun Jim Wu, Wenqing Wu, Ying-Jiun Hsieh

**Affiliations:** ^1^MBA Program in South East Asia, National Taipei University of Education, Taipei, Taiwan; ^2^College of Management and Economics, Tianjin University, Tianjin, China; ^3^Graduate Institute of Technology Management, National Chung Hsing University, Taichung, Taiwan

**Keywords:** entrepreneurship, dark side, bright side, digital technology, entrepreneurship education

## Introduction

Entrepreneurship, as an important tool for technological innovation, economic development, and social progress, has received unprecedented attention. However, blindly focusing on the positive effects of entrepreneurship will only lead to the failure of entrepreneurship theory construction. As a result, some studies have called on scholars to pay attention to both the bright side and the dark side of entrepreneurship. Existing research has analyzed the impact of the bright side and the dark side of entrepreneurship at the individual level and the social level, respectively. For example, some studies have analyzed the gains and losses faced by entrepreneurs at the individual level, but there is still a gap in the understanding of the likelihood of entrepreneurs experiencing gains and losses and the ways to recover from losses. In addition, some studies have found that entrepreneurship as a creative destruction activity may have positive/negative effects on regions and communities, but there is a significant lack of understanding of the reasons for the different effects. Therefore, this Research Topic has fully analyzed the impact of various entrepreneurial activities on entrepreneurs, new ventures, and regional development in the entrepreneurial context.

The primary goal of this Research Topic is to help scholars and practitioners think more systematically about the bright and dark sides of entrepreneurship. All the papers in this special issue have undergone double-blind peer review. The 11 papers in this special issue contribute to a more systematic understanding of the bright and dark side of entrepreneurship through a variety of theories, methods, and disciplinary perspectives. The following sections will cover digital technology and entrepreneurship, entrepreneurship education, entrepreneurship failure, social entrepreneurship, venture capital, and regional entrepreneurship.

## Digital technology and entrepreneurship

Cai intends to improve the optimization of social media's nature of corporate social responsibility (CSR) and standardize the influence mechanism of social media. First, the research analyzes the concepts of social media, CSR, and corporate reputation from the perspective of entrepreneurial psychology and expounds on the influencing factors of CSR scores from a macro perspective. Second, the mechanism of social media's role in CSR is briefly discussed. On this basis, it is found that the most intuitive manifestation of social media platforms affecting the communication of CSR is the impact on the score of CSR. Finally, 78 listed companies related to the mobile communication industry are selected as samples for the questionnaire survey and statistical analysis, and the hypothesis is demonstrated. The results demonstrate that the hypothesis that the social media platform “WeChat” has an impact on the communication of CSR is valid. It is assumed that the number of user readings and likes is positively correlated with the increase in CSR score, which is valid under a limited sample. It is concluded that the WeChat platform has the best effect on the communication of CSR and can provide the impetus for the improvement of corporate reputation. The correct operation of “social media” will have an impact on the communication of CSR. There is a positive correlation between user reads, likes, and increases in CSR scores. This research helps enterprises to effectively fulfill their social responsibilities and improve the efficiency of CSR. This makes up for the lack of social media's influence mechanism on the nature of CSR. It innovatively explores the impact of social media on CSR from the perspective of entrepreneurial psychology and provides some ideas for entrepreneurs and enterprises to create CSR and CSR value.

Liu et al. aim to explore the impact mechanism of the new dimension of entrepreneurship on the innovation performance of enterprises in the context of digital transformation. The relationship between entrepreneurship and enterprise innovation performance is studied in Beijing, Shanghai, Guangzhou, Shenzhen, and other high-tech enterprises represented by innovative companies. Firstly, the correlation hypothesis between variables is proposed through the induction and summary of relevant data and academic achievements. Statistical data is collected using a questionnaire survey. Finally, the proposed hypothesis is verified through regression analysis. The correlations between innovative spirit, adventurous spirit, integrity, and enterprise survival performance are positive. It shows that entrepreneurship and its three dimensions have a significant positive impact on enterprise entrepreneurial performance. The research examines the logical relationship and influence mechanism between the entrepreneurial spirit of entrepreneurs and the innovation performance of enterprises, which has a certain guiding role in the management practice of innovative enterprises in China.

Li et al. deeply discussed the influence of the media diversification model and entrepreneurship on enterprise financial performance. Besides, they expounded the relevant theories such as the media diversification model and entrepreneurial spirit. Furthermore, Time Publishing & Media is taken as the representative of the media diversification model. Finally, the influence of entrepreneurship on financial performance is discussed regarding entrepreneurship in the Yangtze River Delta as the research object. The profitability, solvency, and operation ability of Time Publishing & Media are analyzed. It is found that there are problems in the profitability and operation ability of Time Publishing & Media. The solvency is good, and the risk of debt repayment is low. As a result, a diversified management model may not have a positive impact on enterprise performance. In addition, the entrepreneurial spirit of the Yangtze River Delta is studied, and the results reveal that the stronger the entrepreneur's ability to take risks, the better the financial performance of the enterprise, but the risk should be appropriate. Therefore, the research on the influence of the media diversification model and entrepreneurship on the financial performance of enterprises in the environment of sustainable development has guided significance for enterprises to improve their business performance and market competitiveness.

Driven by the development of new media, the Internet celebrity economic marketing model has gradually become one of the mainstream online marketing models. It has aroused warm attention on the network platform and provided a breakthrough for entrepreneurship for college students. Xiang and Wang aim to explore the influence of the Internet celebrity economy on college students' entrepreneurial values and entrepreneurial behavior. A questionnaire is conducted among students in two colleges in Xi'an. Moreover, a theoretical model is constructed according to the influence principle of entrepreneurial values on entrepreneurial behavior. The results of the questionnaire show that contemporary college students generally pay attention to Internet celebrities mainly through live broadcast platforms and shopping platforms, among which entertainment and shopping account for the largest proportion. More than 40% of college students are optimistic about the impact of the Internet celebrity economy and remain rational and objective on the whole. The results of the model analysis show that entrepreneurial values have a positive and significant impact on entrepreneurial behavior. The entrepreneurial intention has an incomplete intermediary effect in the influence mechanism of the Internet celebrity economy on entrepreneurial behavior. The chain double intermediary composed of entrepreneurial motivation and entrepreneurial intention has an incomplete intermediary effect in the indirect impact path of the Internet celebrity economy on entrepreneurial behavior. Moreover, entrepreneurial policy satisfaction has a regulatory effect on the impact path of entrepreneurial intention on entrepreneurial behavior. The research results can guide college students to view the Internet celebrity economy rationally and objectively, and provide some guidance for them to have correct entrepreneurial values.

## Entrepreneurship education

Sun et al. aim to promote the organic integration of innovation and entrepreneurship education and art education, further promote the reform of college Students' cultural and aesthetic education, improve college Students' aesthetic perception ability, and help contemporary colleges establish a correct political morality. This thesis aims to further promote the reform of college Students' cultural and aesthetic education, improve college Students' aesthetic perception ability, and help contemporary colleges establish correct political and moral values. First, the connotation of college Students' aesthetic education and the definition of cultural aesthetics are introduced, which is based on the characteristics of two-way interaction, multiple selectivities, timeliness, and popularization of film and television media in the new media era; then, the way of the questionnaire is adopted. With five universities as the research object, 250 questionnaires are distributed, and 235 valid questionnaires are collected, with a valid response rate of 94%. Finally, through the six questions, it is concluded that 68.9% of the students watch 3–5 h a day, and 4.3% of the students watch more than 7 h; 89.4% of the students hold that the same products as stars in film and television will exert an impact on consumption. Film and television culture and art have a positive and negative impact on college Students' cultural aesthetic perception. The positive impact is that the film and television media not only provides a good way to cultivate the aesthetic perception ability of contemporary college students, but also help them to establish the correct aesthetic values. The negative impact is mainly reflected on two levels, namely, the vulgarization of film and television media works and the consumption of aesthetic concepts. The advantage of this exploration is to put forward the reform measures of college Students' cultural and artistic aesthetic education under the current educational background in China to help colleges better carry out college Students' cultural and artistic aesthetic education. Based on this, the reform measures of college Students' cultural aesthetic education under the current education in China were put forward, to help colleges and universities better carry out college Students' cultural aesthetic education.

Cultivating the entrepreneurship of college students is not only a response to the national call, but also a basic requirement for implementing quality education and promoting the comprehensive development of college students. Firstly, Yang et al. analyze the impact of artificial intelligence (AI) technology on social ethics. Secondly, they analyze the current situation of the cultivation of science and engineering college students' entrepreneurship from three aspects: Chinese traditional cultural thoughts influence the concept of career choice, enterprises emphasize utilitarianism, and colleges and universities attach importance to knowledge education and despise spiritual education. Finally, the data statistics on the cultivation of entrepreneurship of science and engineering college students are carried out in the form of questionnaires. The results demonstrate that among the students surveyed, 21.31% have a strong willingness to start their own business, and 72.84% have the idea of starting their own business, which means that most students still want to start a business through their efforts, not blindly looking for jobs. Simultaneously, among many majors, 87.5% of students majoring in agriculture and medicine are better at finding new ways to solve problems, while the proportion of students majoring in literature and history is the lowest. It also indicates that most students believe that schools should add more seminar courses, internship courses, design courses, experimental courses, etc., and allow students to choose learning courses across colleges and majors, to cultivate college students' entrepreneurship. The proposed method provides some ideas for the application of AI technology in the cultivation of students' entrepreneurship.

Qu et al. aim to coordinate the relationship between innovation and entrepreneurship education (IEE) and professional education. This exploration is based on the entrepreneurial spirit of young entrepreneurs and the re-integration of IEE and music education in colleges. First, the IEE is studied in theory. Then, the basic criteria for integrating IEE and professional education are studied, and 305 students from a music college in Xi'an are taken as the survey sample. The questionnaire is adopted to investigate the current situation of the integration of IEE and professional education. The results show that 52.1% of students believe that IEE is closely related to professional education. In terms of self-entrepreneurship awareness, males' awareness of self-entrepreneurship is higher than females', and the willingness for self-entrepreneurship from freshman to senior is 3.1, 15.5, 26.1, and 30.8%, respectively. For the dominant position in the integrated curriculum, 55.6% hold that professional courses should dominate innovative professional courses, and 25.9% believe that innovation and entrepreneurship courses should be dominated. Besides, 16.5% think that the proportion of the two should be the same, and 2% hold that it does not matter. For the enthusiasm for innovative professional courses, only 14.1% of students are very positive. The survey results show that the integration of IEE and professional education needs to be improved, and there is a lack of pertinence and guidance for students of different genders and grades. Students are not clear about the position of IEE and lack enthusiasm. Finally, reasonable suggestions are put forward given the above problems. The results are conducive to promoting and accelerating the process of talent training mode combining professional education and IEE. It has a certain reference value for college education and teaching reform.

## Entrepreneurship failure

There are many reasons for entrepreneurs to start a business, but there is only a thin line between success and failure, and not everyone is willing to try to start a business again after encountering a failure. Therefore, it is worth exploring how start-up losers accumulate the energy of entrepreneurship and the reasons for starting a business again. Pan et al. adopted the typical sampling method to select a suitable and representative case company entrepreneur for an in-depth interview. The results of this study revealed that in the process of the Entrepreneur starting a business three times, the Entrepreneur's personal motivation and learning ability in the face of failure, coupled with family support, made the Entrepreneur willing to keep trying, even though he had to face the risk of repeated entrepreneurial failures, so that he could keep his positive energy on his entrepreneurial journey and eventually achieve a successful outcome.

## Social entrepreneurship

Using core self-evaluation theory, Xiabao et al. assess the effects of internal work locus of control and bricolage on social entrepreneurship orientation. They adopted the cross-sectional survey design using a sampling frame to engage 400 top executives of social enterprises in mainland China. Three hundred and seventy-two of the executives replied, presenting a response rate of 93%. Results of structural equation modeling analysis show significant positive relationships between internal work locus of control, bricolage, and social entrepreneurship orientation. The positive mediating effect of bricolage on the relationship between internal work locus of control and social entrepreneurship orientation was also found to be true. Consequently, to foster social entrepreneurship orientation, top executives of social enterprises need to gather available resources for bricolage tasks. These findings contribute new knowledge to how internal work locus of control affects social entrepreneurship orientation through the bricolage activity of Chinese social enterprises. Through core self-evaluation theory, they demonstrate the effect of internal work locus of control as a preceding factor in the relationship between bricolage and social entrepreneurship orientation.

## Venture capital

Feng et al. find that research and development (R&D) may not always benefit entrepreneurial firms. They focus on the double-edged effect of R&D activities on attracting institutional investment in entrepreneurial firms. Based on a panel dataset of 700 listed entrepreneurial firms in ChiNext, they document: (1) an inverted-U relationship between R&D intensity and future institutional investment, which they argue is evidence that institutional investors are concerned about R&D overinvestment; (2) an inverted-U relationship between R&D capitalization and future institutional investment, which they argue shows suspicion of the institutional investors toward high R&D capitalization. Furthermore, by splitting institutional investors into venture capitals (VCs) and non-venture capitals (non-VCs), they confirm that VCs have higher acceptance of both R&D intensity and capitalization as VCs have more expertise to alleviate a certain level of risks.

## Regional entrepreneurship

Guo et al. select socioeconomic data related to 146 prefecture-level cities included in nine city clusters from 2014 to 2018 to establish a city-level socioeconomic system in China. A sensitivity analysis of regional entrepreneurship and economic quality development based on system dynamics was conducted to explore the changes in regional entrepreneurship and economic quality development over time and their sensitivity factors. In this way, the dynamic evolution mechanism of the system can be portrayed, and the optimization of the system can be achieved through the coordination of the factors within the system. The article sets up three scenarios to explore the fluctuations in regional entrepreneurship and economic quality development when three sensitive factors, namely, business environment, financial services scale, and innovation environment, change. There are differences in the development of cities within city clusters. The business environment and high-quality economic development of the central cities within the city cluster are stronger than those of the non-central cities. Therefore, regions should focus on synergistic development within city clusters when formulating related policies. The variation of regional entrepreneurship development and economic quality development, after a factor in the system, is changed, is asymmetric. Because the sensitivity of different urban clusters and the way they are affected by sensitive factors varies, the state should pay more attention to the adaptability of cities when formulating corresponding policy measures and adapt its policy measures to the sensitivity characteristics of each region according to local conditions.

## Conclusion

In summary, the 11 papers in this special issue aim to understand the impact of various types of entrepreneurial activities on entrepreneurs, new ventures, and regional development. Research Topics include digital technology and entrepreneurship, entrepreneurship education, entrepreneurial failure, social entrepreneurship, venture capital, and regional entrepreneurship. We thank all the authors for their contributions to this Research Topic and all the reviewers for their voluntary contributions to enhancing the quality of the research during the peer review process. Finally, we hope that more scholars will pay attention to the dark and bright side of entrepreneurship in the future, to provide valuable insights for promoting the construction of entrepreneurship theory.

## Author contributions

YW and WW conceived of the idea and coordinated the Research Topic. YW, WW, and Y-JH carried out support tasks for the coordination of the Research Topic and edition of articles. All authors contributed to the article and approved the submitted version.

## Funding

This work was supported by National Science and Technology Council, Taiwan (111-2410-H-003−072-MY3), the National Social Science Foundation of China (Grant No. 21BGL061), The First Batch of New Liberal Arts Research and Reform Practice Projects of the Ministry of Education of China (Grant No. 2021120009), Key Project of Tianjin Philosophy and Social Science Planning (Grant No. TJGL20-026), and Major Social Science Project of Tianjin Education Commission (Grant No. 2019JWZD33).

## Conflict of interest

The authors declare that the research was conducted in the absence of any commercial or financial relationships that could be construed as a potential conflict of interest.

## Publisher's note

All claims expressed in this article are solely those of the authors and do not necessarily represent those of their affiliated organizations, or those of the publisher, the editors and the reviewers. Any product that may be evaluated in this article, or claim that may be made by its manufacturer, is not guaranteed or endorsed by the publisher.

